# Digital psychosocial intervention for depression among older adults in socioeconomically deprived areas in Brazil (PRODIGITAL-D): protocol for an individually randomised controlled trial

**DOI:** 10.1186/s13063-022-06623-z

**Published:** 2022-09-07

**Authors:** Carina Akemi Nakamura, Marcia Scazufca, Felipe Azevedo Moretti, Thiago Vinicius Nadaleto Didone, Mariana Mendes de Sá Martins, Luara Aragoni Pereira, Caio Hudson Queiroz de Souza, Gabriel Macias de Oliveira, Marcelo Oliveira da Costa, Marcelo Machado, Evelyn da Silva Bitencourt, Monica Souza dos Santos, Jamie Murdoch, Pepijn van de Ven, Nadine Seward, William Hollingworth, Tim J. Peters, Ricardo Araya

**Affiliations:** 1 Departamento de Psiquiatria, Faculdade de Medicina FMUSP, Universidade de Sao Paulo, Sao Paulo, Brazil; 2 Instituto de Psiquiatria, Hospital das Clinicas HCFMUSP, Faculdade de Medicina, Universidade de Sao Paulo, Sao Paulo, Brazil; 3 Debasé Audiovisual, Sao Paulo, Brazil; 4 Faculdade de Arquitetura e Urbanismo FAU, Universidade de Sao Paulo, Sao Paulo, Brazil; 5 Department of Population Health Sciences, King’s College London, London, UK; 6 Health Research Institute, University of Limerick, Limerick, Ireland; 7 Health Service and Population Research, Institute of Psychiatry, Psychology and Neuroscience, King’s College London, London, UK; 8 Health Economics Bristol, Population Health Sciences, Bristol Medical School, University of Bristol, Bristol, UK; 9 Population Health Sciences, Bristol Medical School, and Bristol Dental School, University of Bristol, Bristol, UK

**Keywords:** Randomised controlled trial, Process evaluation, Depression, Digital intervention, Psychosocial intervention, Behavioural activation, Psychoeducation, Low- and middle-income countries, Brazil

## Abstract

**Background:**

Depression in older adults is a challenge for health systems in most low- and middle-income countries (LMICs). Digital strategies for the management of this condition have been emerging worldwide, but the effectiveness of most of them is still unclear, especially among older adults. Thus, we aim to assess the effectiveness and cost-effectiveness of a digital psychosocial intervention to treat depression among older adults living in socioeconomically deprived areas in Guarulhos, Brazil.

**Methods:**

We will conduct a two-arm individually randomised controlled trial with 1:1 allocation ratio. Five hundred older adults aged 60 years or over with depressive symptomatology (9-item Patient Health Questionnaire score, PHQ-9 ≥ 10) and registered with one of the primary care clinics will be recruited to participate in this study. A 6-week digital psychosocial programme, named Viva Vida, will be delivered via WhatsApp to participants allocated to the intervention arm. The Viva Vida will send psychoeducational and behavioural activation audio and visual messages 4 days a week for 6 weeks. The control arm will only receive a single message with general information about depression. The primary outcome will be the proportion of depression recovery (PHQ-9 < 10) assessed at 3 months. The cost-effectiveness of the intervention will be assessed at 5 months. A detailed process evaluation will be used to explore context and important implementation outcomes.

**Discussion:**

This programme was based on the PROACTIVE intervention and designed to be delivered without face-to-face contact. If effective, it could be a simple treatment option, appropriate not only when social distancing is required, but it could also be included as a regular public health programme to initiate depression treatment, particularly in LMICs where resources allocated to mental health are scarce.

**Trial registration:**

Registro Brasileiro de Ensaios Clínicos (ReBEC), RBR-4c94dtn. Registered on 22 October 2021 (submitted on 03 August 2021).

## Administrative information

Note: the numbers in curly brackets in this protocol refer to SPIRIT checklist item numbers. The order of the items has been modified to group similar items (see http://www.equator-network.org/reporting-guidelines/spirit-2013-statement-defining-standard-protocol-items-for-clinical-trials/).

**Table:** 

Title {1}	“Digital psychosocial intervention for depression among older adults in socioeconomically deprived areas in Brazil (PRODIGITAL-D): protocol for an individually randomised controlled trial”
Trial registration {2a and 2b}.	Registro Brasileiro de Ensaios Clínicos (ReBEC), RBR-4c94dtn.
Protocol version {4}	Version 5.0, 16 March 2022.
Funding {4}	This study was funded by São Paulo Research Foundation (FAPESP, process number 2017/50094-2) and the Joint Global Health Trials initiative jointly funded by Medical Research Council, Wellcome Trust, and the UK Department for International Development (MRC, process number MR/R006229/1).
Author details {5a}	(1) Departamento de Psiquiatria, Faculdade de Medicina FMUSP, Universidade de Sao Paulo, Sao Paulo, Brazil (2) Instituto de Psiquiatria, Hospital das Clinicas HCFMUSP, Faculdade de Medicina, Universidade de Sao Paulo, Sao Paulo, Brazil (3) Debasé Audiovisual, Sao Paulo, Brazil (4) Faculdade de Arquitetura e Urbanismo FAU, Universidade de Sao Paulo, Sao Paulo, Brazil (5) Department of Population Health Sciences, King’s College London, London, UK (6) Health Research Institute, University of Limerick, Limerick, Ireland (7) Health Service and Population Research, Institute of Psychiatry, Psychology and Neuroscience, King’s College London, London, UK (8) Health Economics Bristol, Population Health Sciences, Bristol Medical School, University of Bristol, Bristol, UK (9) Population Health Sciences, Bristol Medical School, and Bristol Dental School, University of Bristol, Bristol, UK
Name and contact information for the trial sponsor {5b}	Sponsors: Universidade de Sao Paulo (USP), Brazil King’s College London (KCL), United Kingdom Principal Investigators contact: Dr Marcia Scazufca, Universidade de Sao Paulo, Brazil scazufca@usp.br Professor Ricardo Araya, King’s College London, United Kingdom ricardo.araya@kcl.ac.uk
Role of sponsor {5c}	The sponsors and funders have no roles in the design and conduction of the study, data analysis and interpretation, or in the writing or submission of the manuscript for publication.

## Introduction

### Background and rationale {6a}

Depression is a common but often neglected mental health disorder, especially among individuals living in socioeconomically vulnerable situations. Low- and middle-income countries are the most affected by this condition, where health systems are not able to meet high demands for mental health services due to scarce specialised healthcare professionals and economic resources [1, 2]. The demographic changes that LMICs are experiencing add another layer of concern, as it is known that depression at older ages has a high prevalence [2] and is associated with increased healthcare utilisation and costs [3].

The World Health Organization (WHO) recommends the management of depression in primary healthcare, as it is affordable and cost-effective [4]. Based on collaborative care and task sharing principles, our group developed the PROACTIVE intervention, a 17-week programme for the treatment of depression among older adults registered with primary care clinics in Brazil [5]. Short, animated videos were shown during home sessions led by community health workers. The videos used psychoeducation and behavioural activation techniques to teach individuals to recognise their depressive symptoms and to increase the number of meaningful and pleasant activities in their daily lives. PROACTIVE used a dedicated tablet computer application to structure intervention sessions, present multimedia content to trial participants, collect data, and allow communication between stakeholders [6]. Due to the nature of the programme and our target population, the PROACTIVE home sessions were no longer feasible when the COVID-19 pandemic started. Therefore, we designed a remotely delivered intervention (Viva Vida) that will be evaluated in a separate individually randomised controlled trial (RCT) (PRODIGITAL-D) described in this protocol.

Alongside many challenges brought by the pandemic to primary care services, social distancing measures have also highlighted the need for developing evidence-based and cost-effective interventions that do not require primary care professionals or mental health specialists to conduct personal consultations. The development of digital health promotion programmes for mental health has become popular in recent years with the penetration of smartphones throughout the world [7]. Evidence of the effectiveness of digital interventions for depressive symptoms is beginning to emerge from LMICs [8], including Brazil [9]. Nevertheless, interventions targeting older populations have been less explored [10]. We are therefore investigating a 6-week digital ‘psychosocial’ intervention delivered by automated WhatsApp messages to treat depression among older adults in Guarulhos, Brazil.

### Objectives {7}

The objective of the PRODIGITAL-D study is to investigate the effectiveness and cost-effectiveness of treating older adults with depression by a 6-week digital psychosocial intervention (Viva Vida) delivered by WhatsApp messages, compared with a single message. We also aim to evaluate key implementation outcomes and how they influenced recovery from depression.

### Trial design {8}

This is a two-arm, superiority individually RCT with 1:1 allocation ratio and embedded economic and process evaluations.

## Methods: participants, interventions, and outcomes

### Study setting {9}

The study will recruit individuals registered with primary care clinics, known as Unidades Básicas de Saúde (UBS), in Guarulhos, Brazil. Guarulhos is the second most populous city of the state of São Paulo and marked by socioeconomic inequalities. The population is estimated to be 1.4 million, with 39 UBSs working to the Family Health Strategy model spread across four health districts. The 24 largest UBSs will participate in this study.

### Eligibility criteria {10}

#### Inclusion criteria

- Individuals aged 60 or over
- Individuals registered with any of the participating UBSs
- Individuals able to receive and listen to WhatsApp messages
- Individuals screening positive for depressive symptomatology on the Patient Health Questionnaire (PHQ; scores of PHQ-2 ≥ 1 and PHQ-9 ≥ 10) [11]

#### Exclusion criteria

- Individuals with communication issues (non-Portuguese speaking, cognitively impaired or other problem hindering communication to engage in trial assessments or intervention, such as vision or hearing problems)
- Individuals unable to engage in the study for the total period of five months (terminal illness or partner with terminal illness, other)
- Individuals presenting acute suicidal risk (i.e. reported suicidal attempt in the two weeks prior to the screening assessment, as assessed by the 9th item of the PHQ-9 and the Immediate Suicide Risk Protocol)
- Individuals living in the same household as another participant in the study
- Individuals who participated in the PROACTIVE trial

### Who will take informed consent? {26a}

Verbal informed consent will be sought by the research assistant before screening and qualitative assessments and when inviting the eligible individuals to participate in the study. All assessments will be conducted by phone. Verbal consent and assessments will be recorded whenever authorised by the individual.

### Additional consent provisions for collection and use of participant data and biological specimens {26b}

No additional consent for collection and use of participant data and biological specimens will be collected.

## Interventions

### Explanation for the choice of comparators {6b}

Participants will receive a digital psychosocial intervention delivered through the WhatsApp application. The control group will receive a single audio message. This was chosen as it is the closest to the usual practice, and we do not anticipate any major additional therapeutic effect. The research team will not interfere with any other health care participants receive in either arm.

### Intervention description {11a}

#### Digital psychosocial intervention

Participants allocated to the intervention arm will receive the 6-week Viva Vida digital psychosocial programme delivered via WhatsApp messages. The contents of this programme are based on the PROACTIVE intervention [5] and will include psychoeducation about depression and health promotion guidelines, simple ways to solve day-to-day problems related to depressive symptoms, and behavioural activation. Such an approach is anchored in recommendations by the WHO in guides containing step-by-step orientation for digital interventions targeting depression [12]. The proposed intervention is also based on concepts of interactive applications of health communication [13, 14]. Participants will receive approximately 48 audio and visual messages, delivered 4 days a week in the morning and afternoon over a 6-week period. The duration of six weeks was chosen as an appropriate timeframe to ensure sustained participation throughout the whole period.

The audio messages have on average 3 min and use the technique of storytelling, which is a powerful communication tool when used to share and create bonds with other individuals. This technique can involve, convince, remind, and motivate. It can also improve the attention and retention of important information by the target audience. By creating empathy and interest in a story, we can encourage new behaviours and reflections on personal problems.

Once a week, participants will be invited to share their opinions about the programme by responding to a question through the WhatsApp ‘quick reply’ tool, with a up to three-answer option (e.g. yes, more or less, no). After answering the question, participants will also be invited to record and send audio messages to share their experience with the programme. Messages sent by the participants will be answered by automated reply only, as this programme was not designed to reply to individual demands. However, during the intervention, participants will receive messages advising them that they can visit health professionals to receive further advice and care for depression if they feel they need additional support.

#### Single message

Participants allocated to the control group will receive a single audio message with psychosocial contents about depression, such as the main signs of depression and simple ways to improve their mood. Participants will also be advised to talk to health professionals if they do not feel better.

#### System to deliver the messages

During the PROACTIVE RCT, 68% of 2246 screened older adults who owned a mobile phone reported sending and/or receiving messages by WhatsApp. Thus, considering WhatsApp is the most used messaging application in Brazil and it is user-friendly for older adults, a web system integrated into the WhatsApp Business API (Application Programming Interface) was developed to deliver the messages for PRODIGITAL-D participants. The PHP-based Laravel Framework was used in a Linux environment to create the web system, with Apache used to implement the server component and the data retained in a MySQL database. The system is hosted on a cloud service (Cloudways) where the access is restricted using both authentication and authorisation processes. Data flowing to and from browsers are also protected by the use of encryption through Secure Socket Layer certificates. An intermediate company was contracted, as the direct usage of the WhatsApp Business API is restricted to medium and large businesses. The WhatsApp Business API enables us to manage and deliver messages to a large number of participants and to collect information, such as date and time when each message was delivered and visualised, timestamp and content of incoming messages, and participants’ interaction with WhatsApp features (e.g. the aforementioned quick replies).

The intermediate company’s platform is used to register templates of the messages to be sent during the intervention. These templates are approved by the WhatsApp platform prior to first use. They allow messages to be personalised with the name of each participant, the attachment of appropriate media file (audio/image) and other automation processes.

Messages will be scheduled weekly according to the lists of randomised participants and delivered using a ‘cron service’ (job scheduler) on the server, in which commands or scripts are set to run periodically at fixed times, dates or intervals. A dashboard was also developed to track the communication pattern (that is, status of the WhatsApp messages—sent to the intermediate company’s platform, delivered to participants, and opened by participants) and the general performance of the scheduled lists to help us in identifying and following up participants with potential technical problems.

### Criteria for discontinuing or modifying allocated interventions {11b}

After the first 2 weeks of receiving the Viva Vida programme (see “Strategies to improve adherence to interventions {11c}” section), participants identified with a communication pattern that might indicate that our WhatsApp number was blocked (consecutive WhatsApp messages not delivered to participants) will be removed from our broadcast list to abide by WhatsApp policy and to keep our system functional. No new intervention messages will be sent, but participants will be contacted for the follow-up assessment, unless they withdraw their consent for follow-up.

### Strategies to improve adherence to interventions {11c}

During the first 2 weeks participants receive the intervention, the research team will actively identify those who might have an issue receiving or opening the messages, based on the status of the WhatsApp messages, and contact them by phone. After this period, no other strategies will be adopted. Participants who have technical difficulties using the application will be able to contact a dedicated phone number to try to solve the issue with support from the research team throughout the whole period of the trial.

### Relevant concomitant care permitted or prohibited during the trial {11d}

Neither ongoing nor new pharmacological or non-pharmacological treatments, nor health appointments, will be prohibited during the trial.

### Provisions for post-trial care {30}

No post-trial care is planned for this study.

### Outcomes {12}

#### Primary outcome

We will assess the proportion of participants recovered from depression with the PHQ-9 [11, 15] (PHQ-9 score < 10), 3 months after receiving the first message (intervention group) or the single message (control group).

#### Secondary outcomes

The proportion recovered at 5 months (PHQ-9 < 10) will assess the maintenance of any earlier clinical gains. The continuous score of the PHQ-9 will also be considered at 3 and 5 months, as well as, anxiety symptomatology assessed with the Generalized Anxiety Disorder-7 (GAD-7) [16] and loneliness assessed with the 3-item University of California, Los Angeles (UCLA) loneliness scale (3-item UCLA) [17].

#### Cost-effectiveness outcomes

Assessment of quality of life with the European Quality of Life five-dimensional questionnaire, five-level version (EQ-5D-5L) [18], and capability wellbeing with the ICEpop CAPability measure for Older people (ICECAP-O) [19] at 3 and 5 months.

#### Implementation outcomes

We will use qualitative methods to assess: (a) the acceptability, appropriateness, and feasibility of the intervention by participants; (b) whether the intervention was received by participants as intended (fidelity); (c) the participants’ contextual barriers and enablers to the implementation of the intervention; and (d) the relationship between implementation outcomes and clinical effectiveness.

### Participant timeline {13}

**Fig. 1:**
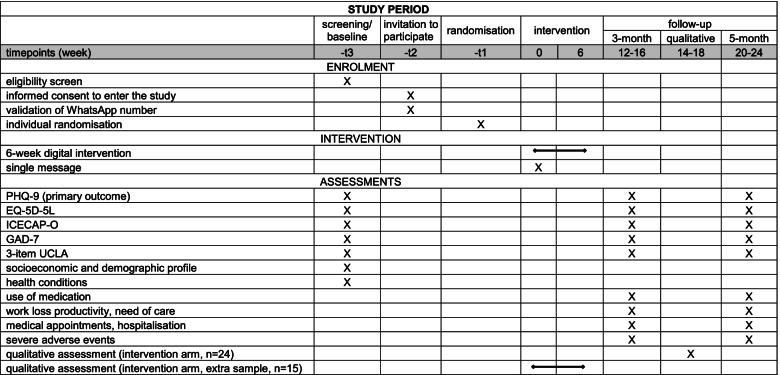
Schedule of enrolment, interventions and assessments. 3-item UCLA, 3-item University of California, Los Angeles (UCLA) loneliness scale; EQ-5D-5L, European Quality of Life five-dimensional questionnaire, five-level version; GAD-7, Generalized Anxiety Disorder-7; ICECAP-O, ICEpop CAPability measure for Older people; PHQ-9, 9-item Patient Health Questionnaire

Participants will be randomised after granting consent to participate in the trial and will be included in a list to start receiving the messages (both control and intervention arms) no more than 10 days after consenting to participate. Each list will include participants recruited during the previous week who will receive the single message (control group) or the first message (intervention group) on the following Monday. Participants allocated to the intervention arm will receive messages every Monday, Wednesday, Friday, and Saturday twice a day during 6 weeks. The follow-up assessment will occur at 3 (weeks 12 to 16) and 5 months (weeks 20 to 24) after receiving the first message. Figure 1 shows the complete timeline of the study.

### Sample size {14}

A sample size of 330–374 individuals will detect a 15 percentage point difference (25% versus 40%) in recovery between the control and intervention groups after 3 months, with 80–85% power and two-sided 5% significance level. Such a difference in recovery rate is considered clinically meaningful [20–24]. We anticipate 25% attrition, which is readily achievable according to the experience we had collecting follow-up data by phone in our previous RCT in Guarulhos, yielding a corrected total sample size of 440–500.

All of the following estimates are based on the PROACTIVE RCT [5] and our pilot study in São Paulo [25]. We expect that on average 10% of the individuals registered with each UBS will be aged 60 years or older. On average, there are approximately 1600 individuals in the eligible age range registered with each UBS. Based on the PROACTIVE data, we would be able to reach 440 individuals by phone and 60% of them (250 individuals) would have a mobile phone and would use WhatsApp application. With a proportion of depressive symptomatology (PHQ-9 ≥ 10) of around 20% [25, 26], there would be 50 individuals potentially eligible in each UBS, though this is likely to reduce to 35 once other entry criteria are applied.

We will work with 24 UBSs, and we plan to complete recruitment in 6 months. We will need to screen approximately 25,000 individuals to achieve a sample of 500 participants. Of these 25,000 individuals, we expect that we will be able to contact 6800. Among the individuals we can contact, 3800 will use WhatsApp. We expect that at least 20% will have depressive symptomatology (*n* = 760), and 530 will consent to participate in the study—that is, at the upper end of the above range from our sample size calculation.

### Recruitment {15}

A list with names and contact details of all individuals aged 59 years or over and registered within the 24 UBSs will be provided by the Health Secretariat of Guarulhos. We will exclude duplicated names and PROACTIVE participants from the list. All individuals in the list will receive a random ID. These IDs will be ordered before entering the study system and the recruitment will follow this order. We will first approach individuals by sending WhatsApp messages to those who have at least one mobile phone number. The message will give a brief introduction to the study and will inform individuals that a research assistant will contact them soon. Initially, only those whose WhatsApp message was successfully delivered will be contacted.

The recruitment comprises three parts: (a) screening, (b) baseline, and (c) invitation to participate in the study. Screening and baseline assessment will be carried out consecutively and individuals will be invited to participate in the study during the same phone call, whenever it is possible. The following are details of each stage of the recruitment process:

a Screening: assessment of inclusion criteria. Before starting the screening, we will inform the participant about the interview and seek consent. Then, we will confirm the age and the use of WhatsApp. Individuals will be screened with the first two questions of the PHQ-9 (PHQ-2). If they present at least one core symptom of depression (depression mood and anhedonia, i.e. PHQ-2 ≥ 1) [27], the evaluation of depressive symptomatology will be completed with the remaining seven questions [11].
b Baseline: Older adults who use WhatsApp, with PHQ-9 ≥ 10 (cut-off for depressive symptomatology) [11] and who do not present acute suicidal risk (as assessed by the 9th item of the PHQ-9 and the Immediate Suicide Risk Protocol) or any other exclusion criteria will complete a full baseline assessment.
c Invitation to participate in the clinical trial: after completing the baseline assessment, the research assistant will provide information about the study (type of interventions and follow-up assessments) for all eligible individuals and invite them to participate in the study. Additionally, the WhatsApp number will be ‘revalidated’ by sending and confirming the receipt of a ‘Welcome’ message.

### Assignment of interventions: allocation

#### Sequence generation {16a}

Participants will be randomly allocated in a 1:1 ratio to either intervention or control arms. Stratification will be based on the PHQ-9 groups (scores of 10–14, 15–19, and 20+), gender (male and female) and age groups (60–69, 70–79 and 80+ years). Random permuted blocks with random block sizes will be used to generate the randomisation sequence with the support of Microsoft Excel.

#### Concealment mechanism {16b}

The randomisation sequence will be concealed in the “randomization module” of the Research Electronic Data Capture (REDCap).

#### Implementation {16c}

The allocation sequence was generated by members of the research team (CAN and TJP) not directly involved in recruiting individual participants, who will be enrolled by an independent research assistant. Data collected will be reviewed by the research coordinator after the participant’s consent to participate in the study and the study arm will be allocated with the support of REDCap. The intervention team will receive the list of randomised participants and will send the allocated messages.

### Assignment of interventions: blinding

#### Who will be blinded {17a}

Given the difference between the number and contents of the messages received by the intervention and control groups, blinding of participants will not be feasible. A list with the participants’ allocation group will be sent every week to the researchers responsible for sending the messages. Research assistants will be completely blind to the group allocation during the follow-up assessments and, whenever possible, the research assistant will not carry out more than one assessment (recruitment and follow-ups) with the same participant. The research teams that coordinate the recruitment and intervention will work separately. The assessments and the interventions will be delivered remotely and individually in order to minimise the risk of contamination.

#### Procedure for unblinding if needed {17b}

No procedure for unblinding is planned for this trial.

### Data collection and management

#### Plans for assessment and collection of outcomes {18a}

Recruitment and the 3- and 5-month follow-up interviews will be carried out by independent research assistants through phone calls. The research assistants will be trained to conduct the assessments and to use the data collection software (REDCap). Quality control of a sample of recorded assessments will be conducted by an independent research assistant. Qualitative assessments will be carried out while participants are receiving the intervention and after the first follow-up assessment.

#### Instruments

Depressive symptomatology will be assessed using the PHQ-9 [11]. This instrument was chosen as it is widely used and there is evidence of validity in Brazil [28, 29]. Anxiety symptomatology will be initially screened with the first two questions of the GAD-7 [16] and if GAD-2 ≥ 1 [30], the remaining five questions will be assessed. The 3-item UCLA will be used to assess loneliness [17]. Quality of life will be assessed with the EQ-5D-5L [18] and the ICECAP-O [19]. Level of depressive symptomatology was associated with these two measures in our previous study [31]. Physical and chronic conditions, depression treatment, sociodemographic profile (level of education, personal and household income, marital status, race and living arrangement), alcohol and tobacco use, and use of WhatsApp will also be asked during the recruitment assessment.

During both follow-up assessments, questionnaires completed at baseline will be repeated including the PHQ-9, GAD-7, 3-item UCLA, EQ-5D-5L, and ICECAP-O. Participants will also be asked about admissions to hospital and consultations related to mental healthcare and any ongoing treatment for depression. Additionally, questions about the experience receiving the messages will be included for the intervention arm. For every participant, we will request the electronic health record system data on psychotropic medications and consultations with doctors and nurses during the period they were participating in the trial.

#### Plans to promote participant retention and complete follow-up {18b}

For each follow-up, we will adopt a 4-week window in order to maximise our chances to complete the follow-up assessments. Whenever we have issues with contacting the participant by phone call, we will first send him/her an audio and/or a text message by WhatsApp with a reminder about the assessment. If the participant does not reply to these messages, the UBS manager or an appointed staff member will be contacted to help us reach him/her. If we are unable to find the participant by phone within the first 3 weeks of the follow-up window, a research assistant will attempt to visit the participant at home to complete a face-to-face assessment.

#### Data management {19}

All assessment data will be collected and managed using REDCap [32, 33] hosted at the Hospital das Clínicas da Faculdade de Medicina da Universidade de Sao Paulo. REDCap is a secure, web-based software platform designed to support data capture for research studies, providing (1) an intuitive interface for validated data capture; (2) audit trails for tracking data manipulation and export procedures; (3) automated export procedures for seamless data downloads to common statistical packages; and (4) procedures for data integration and interoperability with external sources.

#### Confidentiality {27}

This study will be compliant to the National Commission of Ethics in Research (Comissão Nacional de Ética em Pesquisa), Resolution 466/2012 security policies. One member of the team will extract the individual’s information from the list provided by the Health Secretariat of Guarulhos, and then they will receive a random ID number. The same ID number will be used when entering data into the REDCap platform and the system developed to deliver the messages. The access to both systems is secured by password and limited according to the members of the research team’s assigned roles and permissions. Audio files of recruitment, follow-up, and qualitative assessments will be renamed using the corresponding ID number and initials and stored on a password-protected platform. Only the recruitment coordination team will have access to these audio files. No paper records will be kept.

#### Plans for collection, laboratory evaluation, and storage of biological specimens for genetic or molecular analysis in this trial/future use {33}

No biological specimens will be collected during this study.

### Statistical methods

#### Statistical methods for primary and secondary outcomes {20a}

The analysis for all outcome measures will follow the intention-to-treat principle and adhere to Consolidated Standards of Reporting Trials guidelines for randomised trials [34]. To understand if there are any imbalances in risk factors for depression, descriptive statistics will be used to compare differences in baseline characteristics between treatment arms.

All outcomes will be evaluated using regression models that adjust for stratification, the baseline assessment of the corresponding outcome measure. For secondary analyses only, any baseline variables that are not balanced between treatment arms will be accounted for in these models. Logistic regression models will be used to evaluate for the primary outcome (recovery from depression at 3 months) and the secondary outcome (recovery from depression at 5 months). Odds ratios with 95% confidence intervals will be used to estimate recovery from depression at 3 and 5 months. Other secondary outcomes including PHQ-9, GAD-7, 3-item UCLA, EQ-5D-5L, and ICECAP-O scores will be assessed using linear regression models. Coefficients and associated 95% confidence intervals for these outcomes represent differences in means between the intervention and control arms. Sensitivity analyses will be conducted to investigate any potential clustering at the USB level.

#### Economic analysis

Two economic analyses will be conducted. (1) Incremental cost-effectiveness between the two arms of the trial will be estimated using (a) the primary clinical outcome measure (cost per participant recovered) and (b) quality-adjusted life years (QALYs) calculated using the EQ-5D-5L [18, 35]. These results will be presented as incremental cost-effectiveness ratios and cost-effectiveness acceptability curves. These show the probability of the intervention being cost-effective at a range of ‘willingness-to-pay’ threshold levels. (2) The net monetary benefit statistic (NMB), using the difference in costs and the difference in QALYs between the two arms, will be calculated at the WHO recommended threshold for LMICs.

We will conduct the cost-effectiveness analysis from the health system perspective to compare the costs and effects of the 6-week digital psychosocial programme against a single message. The EQ-5D-5L [35–37] responses will be converted into utility scores using the population tariff most appropriate for the Brazilian population available at the time of the analysis. Utility scores will be used to estimate QALYs, adjusting for baseline values. We will use national, where available, or local unit costs to value resource use.

#### Interim analyses {21b}

No interim analyses are planned for this trial.

#### Methods for additional analyses (e.g. subgroup analyses) {20b}

A Complier Average Case Effect (CACE) analysis will be used to determine the effect of number of messages listened to on recovery from depression at both 3 and 5 months. A threshold value (listening to most of the messages received) will be used for the CACE analysis with PHQ-9 scores at both time points. We will use this threshold as it was hypothesised to be the minimum number of sessions needed to have a therapeutic effect. A sensitivity analysis using the thresholds of listening to at least half of the messages or all messages will be conducted, and we will also consider using the number of messages opened as a continuous variable unless the relevant relationship is far from linear.

Additional exploratory subgroup analyses will be used to estimate whether the following pre-specified characteristics of the participants modified the effect of the intervention: baseline PHQ-9, gender, age, education level, and presence of co-morbid physical illnesses. To test for modification, an interaction term will be introduced between the pre-specified variable and the treatment arm. The results of these analyses will need to be interpreted with caution due to the limited power to detect such interactions as well as the paucity of evidence on the theoretical basis for these hypotheses.

#### Methods in analysis to handle protocol non-adherence and any statistical methods to handle missing data {20c}

Patterns and the proportion of missing data for the primary and secondary outcomes will be investigated. If there are any marked differences in missing data between treatment arms, or if greater than 10% of the data are missing, multiple imputation using chained equations (MICE models) under the assumption that data is missing at random (MAR) will be used. Data will be imputed separately for the different treatment arms and MICE models will include any variables that predict missingness. Sensitivity analyses testing for modest departures against the MAR assumption will be conducted using the Selection Model Approach.

#### Plans to give access to the full protocol, participant level-data and statistical code {31c}

Participant level-data and statistical code will be available for external use 24 months after publishing the paper with effectiveness results. A research proposal with defined aims and a statistical analysis plan should accompany any request and all requests will be evaluated by the joint principal investigators before granting access to the data.

#### Process evaluation

Two assessments of implementation outcomes will be conducted by phone using qualitative interviews. These interviews will provide insight into the reasons, motivations, modes, and contexts of participants from intervention arm that may affect their clinical and implementation outcomes, as well as the detailed process and content perspectives of how the intervention was received by participants. Approximately 24 participants who received the intervention will be purposively selected for the first assessment, conducted individually, 15 to 30 days after the 3-month follow-up assessment. We will ensure adequate representation from the following subgroups: gender (women and men), age (60–69 years old and 70 years or over), depression status at follow-up 1 (recovered and not recovered from depression). For the second assessment, we will recruit an extra group of 15 individuals using the same inclusion and exclusion criteria of the trial. They will not be included in the main trial analyses so as to eliminate any potential influence these interviews may have on depression outcomes. They will be interviewed weekly by the same research assistant to assess key implementation outcomes throughout the course of the 6-week Viva Vida Programme as well as exploring participants’ contextual and behavioural barriers/enablers to the implementation of the intervention. Informed consent to participate and the qualitative assessments will be audio recorded if the participant allows. Interviews will last around 30 min.

#### Qualitative analysis

All interviews will be audio-recorded, transcribed and analysis will be conducted using Atlas.TI software. The qualitative assessment applied between follow-up assessments will be thematically analysed deductively using pre-established categories (acceptability, appropriateness, fidelity, and feasibility), as well as inductively for other relevant categories emerging from the analysis. In-depth exploration of participants’ experiences will enable the generation of hypothetical propositions about the relationship between clinical outcomes (considering the PHQ-9 score assessed at the beginning of the study and in the follow-up assessment) and implementation outcomes. Analysis of narratives and images collected during the intervention will also be thematically analysed to understand the participant journey (including behavioural activation, contextual barriers, facilitators. etc.) as well as how participants’ experiences changed over the six weeks of receiving the intervention.

## Oversight and monitoring

### Composition of the coordinating centre and trial steering committee {5d}

#### Coordinating centre

Universidade de Sao Paulo, Sao Paulo, Brazil

#### Trial Steering Committee (TSC)

Independent members: Professor Simon Gilbody (University of York, UK); Professor Roberto Alves Lourenço (Universidade Católica do Rio de Janeiro, Brazil); Dr Rodrigo Fonseca Martins Leite (Universidade Municipal de São Caetano do Sul, Brazil); and Paula Verônica Martini Maciel (Escola SUS, Guarulhos Secretary of Health, Brazil).

Trial team members: Dr Marcia Scazufca (Universidade de Sao Paulo, Brazil); Professor Ricardo Araya (King’s College London, UK); and Professor Tim Peters (University of Bristol, UK).

The Trial Steering Committee (TSC) will meet once a year, either virtually or in person.

### Composition of the Data Monitoring Committee, its role and reporting structure {21a}

#### Data Monitoring Committee (DMC)

Under the guidance of the TSC, a Data Monitoring Committee (DMC) will be formed comprising an independent Chair, an independent statistician (Dr Melissa Newman, London School of Hygiene and Tropical Medicine, UK), one other independent member, and at least one of the trial statisticians (Professor Tim Peters and/or Dr Nadine Seward).

### Adverse event reporting and harms {22}

The risk associated with the trial and the digital intervention are considered minimal. Participants will receive any pharmacological and non-pharmacological treatment they were prescribed before or during the study. Also, participants of both arms will receive messages advising them to seek healthcare if the symptoms are not improving or if they are worsening. Identification of acute suicide risk at recruitment or follow-up assessments will activate a standardised protocol, and a family member and the UBS manager will be contacted by the research team. This protocol was successfully applied in the PROACTIVE study [5, 25]. Individuals identified with an acute suicide risk during the recruitment assessment will not be eligible to participate in the study.

### Frequency and plans for auditing trial conduct {23}

Characteristics used for stratification of randomisation will be monitored in order to ensure that the allocation sequence and REDCap “randomization module” are working as expected, and the two study arms are reasonably well balanced in each stratum. The TSC will be presented with a report of the trial conduct.

### Plans for communicating important protocol amendments to relevant parties (e.g. trial participants, ethical committees) {25}

Modifications on the study protocol will be discussed between the research team, TSC and DMC and submitted to approval of the Ethics Committee. Whenever relevant, Guarulhos health system managers and coordinators will be consulted before any decision.

### Dissemination plans {31a}

Publications on internationally competitive journals and presentation at relevant conferences are expected from this trial. Meetings with the main stakeholders will be organised at the end of the trial to present the main results and discuss further steps and potential future collaboration.

## Discussion

Although there are many barriers for older adults to access digital mental health interventions, they are important strategies for situations when face-to-face contact should be avoided or there is scarcity of health professionals. In our previous study, due to the COVID-19 pandemic, face-to-face sessions were adapted to be delivered by phone as an easy solution for the sudden interruption of home sessions participants had been receiving. An intervention where a trained primary care professional provides a consultation by phone was considered not feasible in most primary care clinics in Brazil. Thus, we have designed a simpler intervention with no health professionals involved. To facilitate access to the proposed intervention, instead of developing a whole new mobile application, we have chosen to use WhatsApp application, as it is widely used in Brazil and more people would be eligible to participate in the study. Also, audio messages based on the animated videos from the previous programme were created to allow individuals with low literacy levels to understand the content of the messages. Importantly, to better understand why or why not the intervention was effective, we are collecting information on key implementation outcomes. To the best of our knowledge, this is the first study evaluating an intervention for older adults with depressive symptoms delivered by WhatsApp. Therefore, if cost-effective, this intervention could be delivered to large number of people and as an affordable first step of a stepped-care intervention to treat depression in primary care.

### Trial status

Protocol version 5.0 of 16 March 2022. The recruitment of the study started in September 2021 and finished in April 2022. Data collection is expected to be completed by October 2022.

## Data Availability

Study documentation and trial data will be available on request according to data sharing conditions.

## Abbreviations

3-item UCLA: 3-Item University of California, Los Angeles (UCLA) loneliness scale
API: Application Programming Interface
CACE: Complier Average Case Effect
DMC: Data Monitoring Committee
EQ-5D-5L: European Quality of Life five-dimensional questionnaire, five-level version
GAD: Generalized Anxiety Disorder
ICECAP-O: ICEpop CAPability measure for Older people
LMIC: Low- and middle-income countries
MAR: Missing at random
MICE: Multiple imputation using chained equations
NMB: Net monetary benefit
PHQ: Patient Health Questionnaire
QALYs: Quality-adjusted life years
RCT: Randomised controlled trial
REDCap: Research Electronic Data Capture
TSC: Trial Steering Committee
UBS: Unidades Básicas de Saúde
WHO: World Health Organization
